# Supplementary material for: Loxl3 Promotes Melanoma Progression and Dissemination Influencing Cell Plasticity and Survival
Source: Cancers (Basel). 2022 Feb 25;14(5):1200. doi: 10.3390/cancers14051200 (PMC8909883; doi:10.3390/cancers14051200)
Supplement: Supplementary file 1 [file cancers-14-01200-s001.zip › cancers-1545076 original figures.pdf]

Supplementary Western blot panels and quantifications

Relative to figure 4B

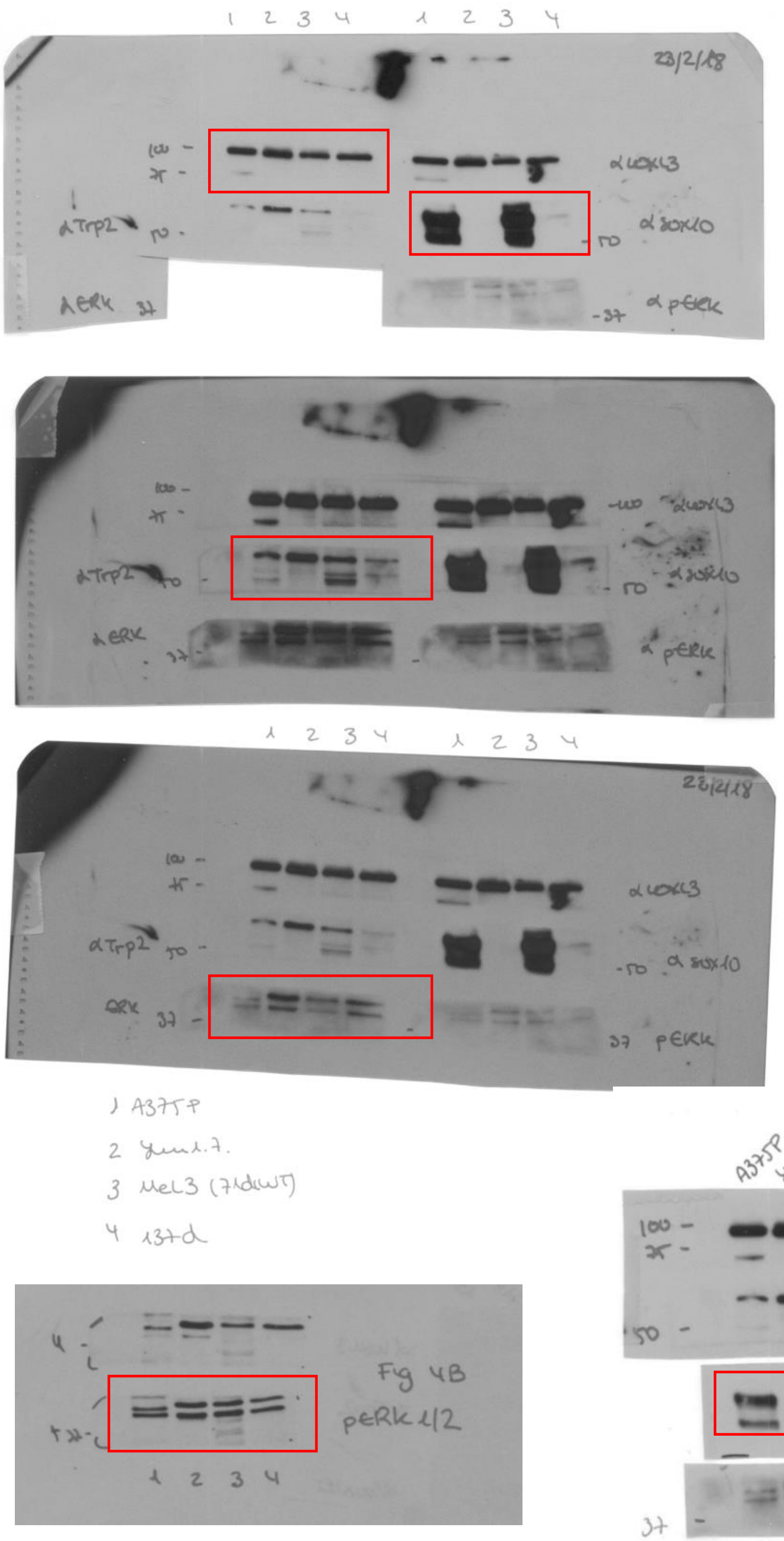

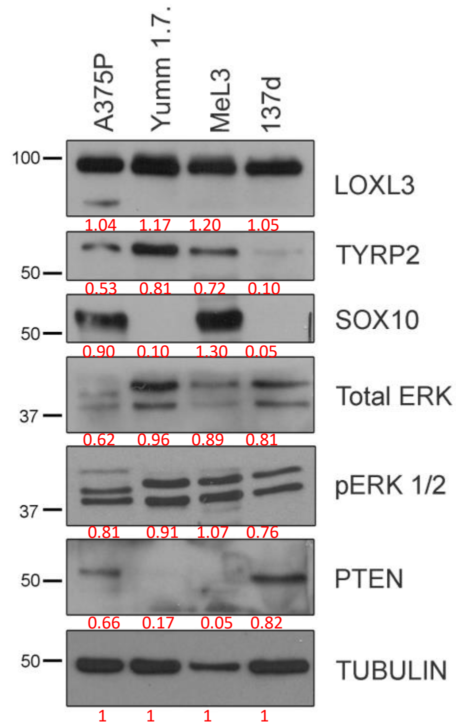

The original scans of the blots are shown on the left and the red squares depict the images used for the final figure shown on the right. The red numbers below each blot panel on the final figure indicate the quantifications of the blot band intensity relative to TUBULIN expression in each condition.

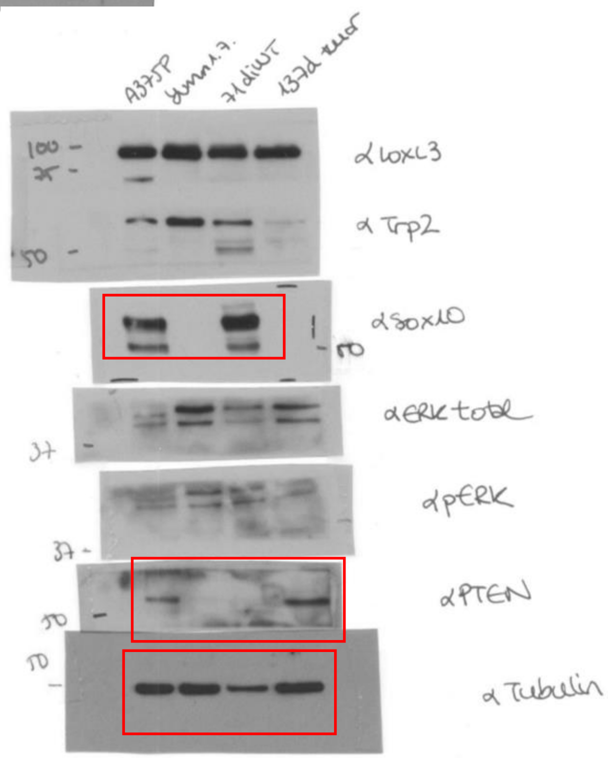

Relative to figure 4D

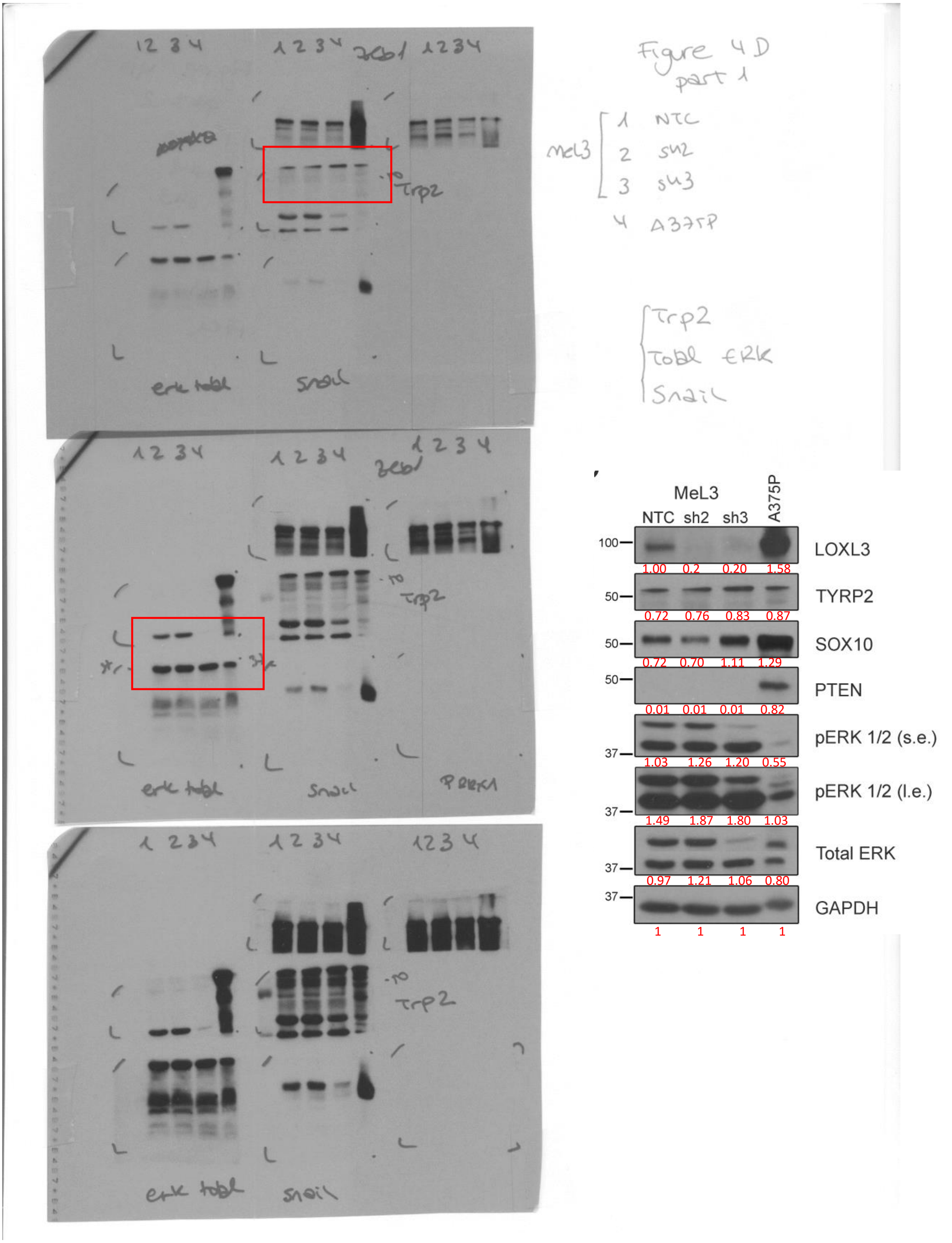

The original scans of the blots are shown on the left and the red squares depict the images used for the final figure shown on the right. The red numbers below each blot panel on the final figure indicate the quantifications of the blot band intensity relative to GAPDH expression in each condition.

Relative to figure 4D

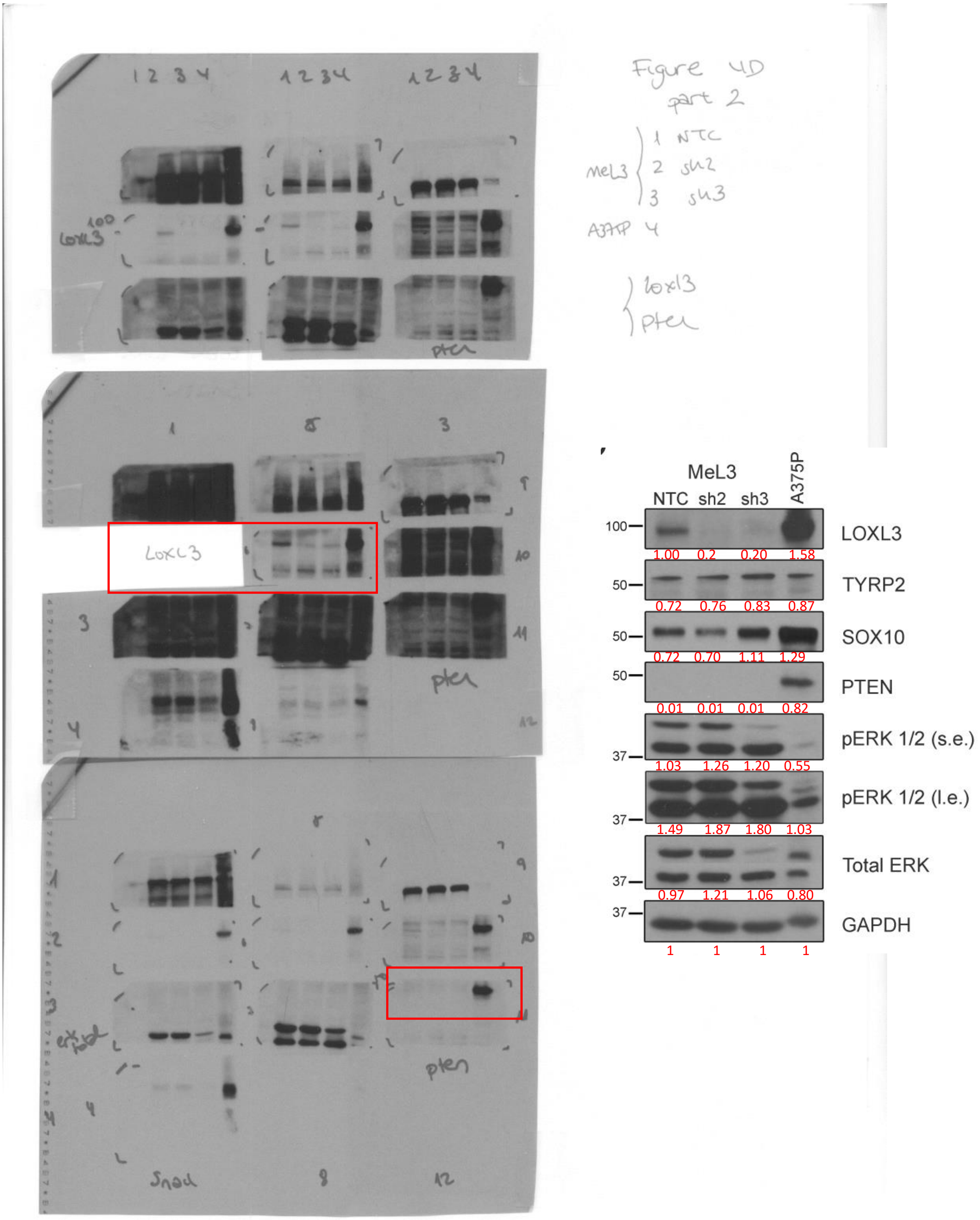

The original scans of the blots are shown on the left and the red squares depict the images used for the final figure shown on the right. The red numbers below each blot panel on the final figure indicate the quantifications of the blot band intensity relative to GAPDH expression in each condition.

Relative to figure 4D

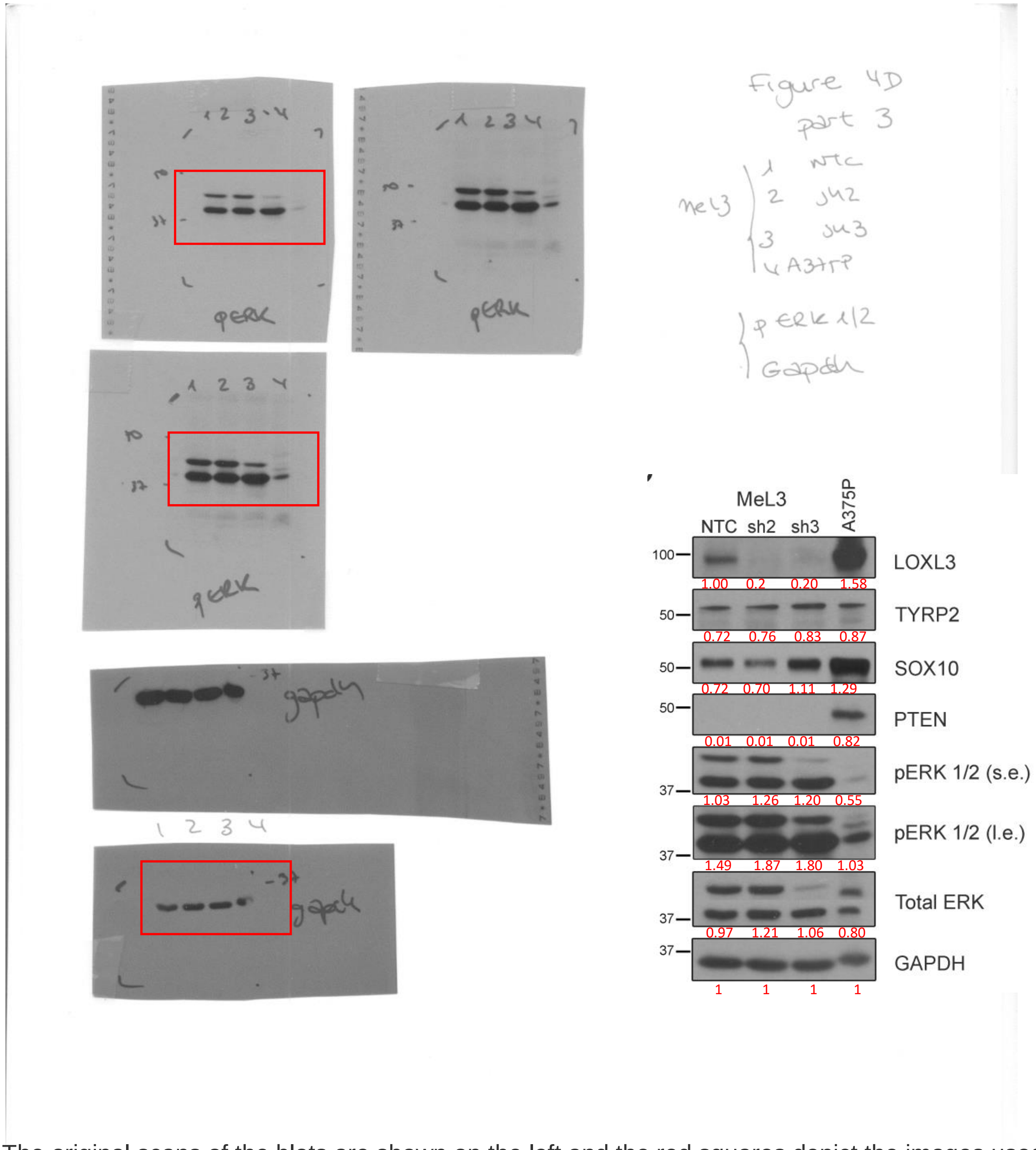

The original scans of the blots are shown on the left and the red squares depict the images used for the final figure shown on the right. The red numbers below each blot panel on the final figure indicate the quantifications of the blot band intensity relative to GAPDH expression in each condition.

Relative to figure 6A

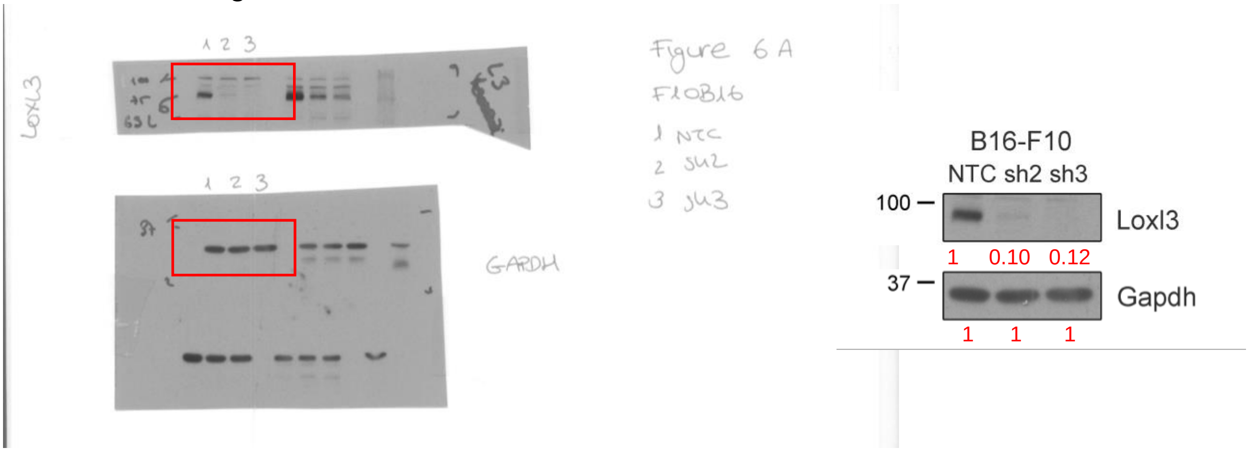

The original scans of the blots are shown on the left and the red squares depict the images used for the final figure shown on the right. The red numbers below each blot panel on the final figure indicate the quantifications of the blot band intensity relative to Gapdh expression in each condition.

Relative to figure 7B

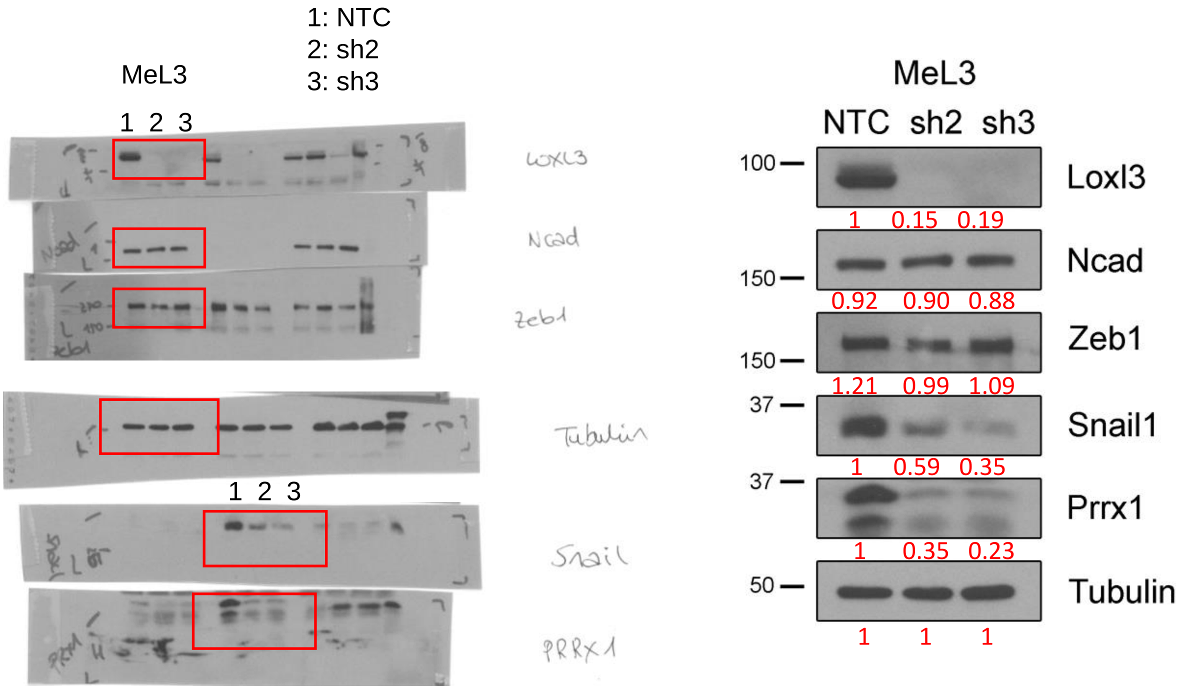

The original scans of the blots are shown on the left and the red squares depict the images used for the final figure shown on the right. The red numbers below each blot panel on the final figure indicate the quantifications of the blot band intensity relative to Tubulin expression in each condition.

Relative to figure 7E

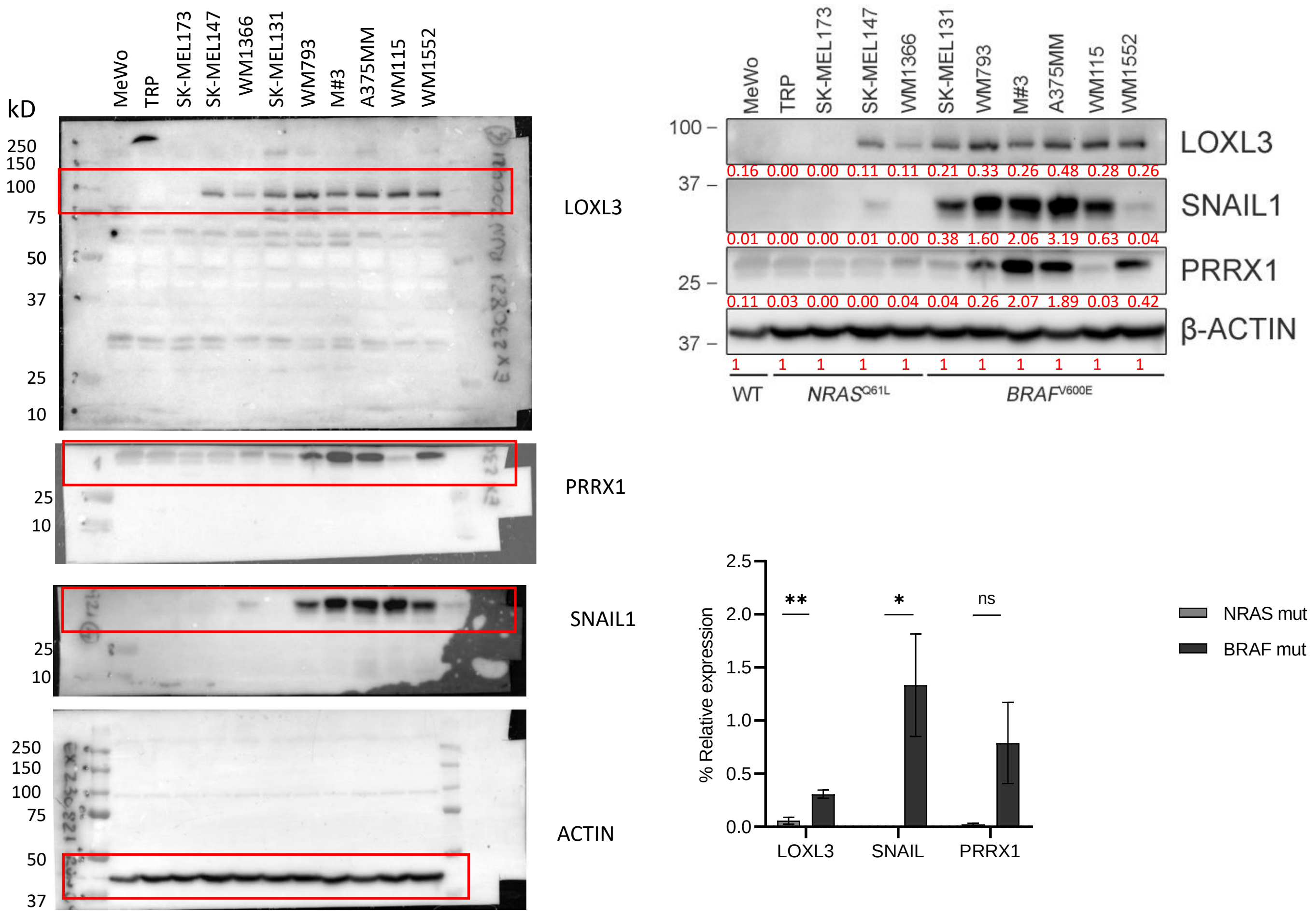

The original scans of the blots are shown on the left and the red squares depict the images used for the final figure shown on the right. The red numbers below each blot panel on the final figure indicate the quantifications of the blot band intensity relative to B-ACTIN expression in each condition.
