# Supplementary material for: Loxl3 Promotes Melanoma Progression and Dissemination Influencing Cell Plasticity and Survival
Source: Cancers (Basel). 2022 Feb 25;14(5):1200. doi: 10.3390/cancers14051200 (PMC8909883; doi:10.3390/cancers14051200)
Supplement: Supplementary file 1 [file cancers-14-01200-s001.zip › cancers-1545076 supplementary.pdf]

Supplementary materials

Supplementary methods

Colony formation assays

To assess anchorage dependent growth, 250 B16-F10 cells were seeded on 6-well plates (Falcon) in DMEM supplemented with 5% FBS, 2 mM L-glutamine and 1% Penicillin-Streptomycin for three weeks. Formed colonies were fixed with 4% parafolmadehyde (Merck) (in PBS 1X) for 20 min and stained with 1% crystal violet (in H_20) for 15 min, washed with H_20 and allowed to dry. For anchorage independent growth assays in semisolid agar, a lower layer of 0.7% agar (Lonza) in culture media is solidified in 12-well plates and 5.000-10.000 cells embedded in culture media with 0.35% agar are seeded on top. After adding 1 ml of culture media, plates were incubated for one week. In both experiments, pictures (Nikon ELWD 0.3/OD 75 camera) were taken in a bright filed microscope (Nikon Eclipse TS100, 4X objective) and colonies were counted using ImageJ software.

Supplementary figure legends

Figure S1. Loxl3 silencing decreases the clonogenic and transformation potential of B16-F10 cells. Formed colonies from control (NTC) and Loxl3-silenced (sh2 and sh3) B16-F10 were counted in anchorage dependent (right) and independent (left) colony assays performed as detailed in supplementary methods. Representative pictures from three independent experiments are shown on the upper panels and the quantifications depicted on the bottom graphs. Mean ± SEM of three independent experiments is shown (A-C). *P*-values were calculated by two-sided unpaired Student´s *t*-test. *p<0.05 and ***p<0.001.

Supplementary tables

Table S1. List of primers used for genotyping and RT-qPCR analyses.

Table S2. List of primary and secondary antibodies used for western blot, immunofluorescence or immunohistochemistry.

Table S1

Table S1: List of primers used for genotyping and RT-PCR analyses

ALLELE/GENE	FORWARD PRIMER	REVERSE PRIMER
Loxl3^{wt}/*Loxl3*^{loxP}	5’-CAGAAAAACGCCACAGGGGAGACG-3’	5’-GCCTCACTCGGCCTTGAGGCTAATC-3’
Loxl3^{LacZ}	5’-CGTCGTTTTACAACGTCGTGAC-3’	5’-TAACAAACCCGTCGGATTCTC-3’
L3^{KO}	5′-CAGAAAAACGCCACAGGGGAGACG-3′	5′-CGGAGTCGCACCTCCTCCACTTGC-3′
Braf^{WT}/*Braf*^{CA}/*Braf*^{V600E}	5’-TGAGTATTTTTGTGGCAACTGC-3’	5’-CTCTGCTGGGAAAGCGGC-3’
Pten/*Pten*^{loxP}/*Pten*^{flox}	5’-ACTCAAGGCAGGGATGAGC -3’	5’-AATCTAGGGCCTCTTGTGCC-3’<br>5’-GCTTGATATCGAATTCCTGCAGC-3’
Cre recombinase	5’-GCCTGCATTACCGGTCGATGC-3’	5’-CAGGGTGTTATAAGCAATCCCC-3’
Prrx1a	5’-ACAGCCTCTCCGTACAGCGC-3’	5’-AGTCTCAGGTTGGCAATGCT-3’
Prrx1b	5’-CATCGTACCTCGTCCTGCTC-3’	5’-GCCCCTCGTGTAAACAACAT-3’
Gapdh	5’-GGTGAAGGTCGGTGTGAACG-3’	5’-CTGCCTCCTGGAAGATGGTG-3’
Loxl3	5’-AGGGGGACTGAAGGAAGTGT-3’	5’-TCCACTTGCAACTGATGCTC-3’
Snail1	5’-AAACCCACTCGGATGTGAAG-3’	5’-AGACTCTTGGTGCTTGTGG-3’
Snail2	5’-CCTTTCTCTTGCCCTCACTG-3’	5’-ACAGCAGCCAGACTCCTCAT-3’
Twist1	5’-CTGCCCTCGGACAAGCTGAG -3’	5’- CTAGTGGGACGCGGACATGG-3’
Mitf	5’-GGGAACAGCAACGAGCTAAG-3’	5’-TGATGATCCGATTCACCAGA-3’
Zeb1	5’-GGGGCATCTCACACTTTTGT-3’	5’-AACGGCTGTGAACCAAAAAC-3’
Zeb2	5’-CCACCAGCCCTTTAGGTGTA-3’	5’-CCCTTGTTCTTCTGGCTGAG-3’
Human Loxl3	5’-TGCTGGAGTCATCTGTTCTGAG-3’	5’-TCTTCGATGTAGGCGGTCTC-3’
Human Prrx1	5’-CTGATGCTTTTGTGCGAGAA-3’	5’-ACTTGGCTCTTCGGTTCTGA-3’
Human L32	5’- GATCTTGATGCCCAACATTGGTTATG-3’	5’- GCACTTCCAGCTCCTTGACG-3’

Table S2

Table S2: List of primary and secondary antibodies used for western blot, immunofluorescence or immunohistochemistry.

PROTEIN	SPECIES [1]	SOURCE	REFERENCE	DILUTION for WB/IF/IHQ [2]
LOXL3	mMab	Santa Cruz	sc-377216	1:200/--/--
S100b	mMab	Santa Cruz	sc-393919	1:500/--/--
SOX10	mMab	Santa Cruz	sc-365692	1:200/--/--
SOX10	gPab	Santa Cruz	sc-17342	--/--/*
α-Tubulina	mMab	Sigma	T5168	1:5.000/--/--
TYRP2	gPab	Santa Cruz	sc-10451	--/--/*
TYRP2	mMab	Santa Cruz	sc-74439	1:500/--/--
PTEN	rPab	Cell Signaling	9552S	1:1000/--/--
GAPDH	mMab	Calbiochem (Merk-Millipore)	CB1001	1:5000/--/--
Snail1	mMab	Cell Signaling	3895	1:300/--/--
SNAIL1 (SN9H2)	ratMab	Cell Signaling	4719	1:1000/--/--
ZEB1	rPab	Sigma-Aldrich	HPA027524	1:500/--/--
Prrx1	mMab	Santa Cruz	sc-293386	1:300/--/--
PRRX1 (Clone: OTI1E10)	mMab	Origene	TA803116	1:2000/--/--
β-Actin	mMab	Abcam	ab6276	1:2500/--/--
y-H2AX (Ser139)	mMab	Millipore	05-636	--/1:300/--
53BP1	rPab	Santa Cruz	sc-22760	--/1:300/--
anti-rabbit-HRP	dkPab	GE Healthcare	NA934	1:3000/--/--
anti-mouse-HRP	sPab	GE Healthcare	NA931	1:3000/--/--
anti-mouse-Alexa-488	gPab	Molecular Probes	A11029	--/1:500/--
anti-rabbit-Alexa-488	gPab	Molecular Probes	A11034	--/1:500/--
anti-mouse-Alexa-546	gPab	Molecular Probes	A11030	--/1:500/--
anti-rabbit-Alexa-647	gPab	Molecular Probes	A21245	--/1:500/--
DAPI	NA	Molecular Probes	D1306	--/1:500/--
anti-mouse HRP	rPab	Dako	P0260	1:5000/--/--
anti-rabbit HRP	gPab	Dako	P0448	1:5000/--/--
anti-rat HRP	rPab	Dako	P0450	1:10000/--/--

[1]**mMab**: Mouse monoclonal antibody; **rPab**: rabbit polyclonal antibody; **rMab**: Rabbit monoclonal antibody; **ratMab**: Rat monoclonal antibody; **gPab**: goat polyclonal antibody. **dkPab**: donkey polyclonal antibody; **sPab**: sheep polyclonal antibody

[2] **WB**: Western-blot; **IF**: Immunofluorescence; **IH**: Immunohistochemistry; **FC**: flow cytometry.

HRP: horseradish peroxidase;

*Available Techniques (Mouse Samples): Antibodies
